# Cellular and molecular events in colorectal cancer: biological mechanisms, cell death pathways, drug resistance and signalling network interactions

**DOI:** 10.1007/s12672-024-01163-1

**Published:** 2024-07-20

**Authors:** Lei Yan, Jia Shi, Jiazuo Zhu

**Affiliations:** 1 Medical Department, The Central Hospital of Shaoyang Affiliated to University of South China, Shaoyang, China; 2 Department of Obstetrics and Gynecology, The Central Hospital of Shaoyang Affiliated to University of South China, Shaoyang, China; 3 Department of Oncology, Xuancheng City Central Hospital, No. 117 Tong Road, Xuancheng, Anhui China

**Keywords:** Colorectal cancer, Gastrointestinal tumors, Genetic and epigenetic factors, Apoptosis, Autophagy

## Abstract

Colorectal cancer (CRC) is a leading cause of cancer-related deaths worldwide, affecting millions each year. It emerges from the colon or rectum, parts of the digestive system, and is closely linked to both genetic and environmental factors. In CRC, genetic mutations such as APC, KRAS, and TP53, along with epigenetic changes like DNA methylation and histone modifications, play crucial roles in tumor development and treatment responses. This paper delves into the complex biological underpinnings of CRC, highlighting the pivotal roles of genetic alterations, cell death pathways, and the intricate network of signaling interactions that contribute to the disease's progression. It explores the dysregulation of apoptosis, autophagy, and other cell death mechanisms, underscoring the aberrant activation of these pathways in CRC. Additionally, the paper examines how mutations in key molecular pathways, including Wnt, EGFR/MAPK, and PI3K, fuel CRC development, and how these alterations can serve as both diagnostic and prognostic markers. The dual function of autophagy in CRC, acting as a tumor suppressor or promoter depending on the context, is also scrutinized. Through a comprehensive analysis of cellular and molecular events, this research aims to deepen our understanding of CRC and pave the way for more effective diagnostics, prognostics, and therapeutic strategies.

## Highlights

- Colorectal cancer (CRC) is one of the leading causes of death among patients.
- CRC has been characterized with changes at the genetic and epigenetic factors.
- The molecular factors can be used as diagnostic and prognostic factors in CRC.
- The cell death mechanisms demonstrate dysregulation in CRC.
- Autophagy has aberrant activation in CRC and exerts dual function.

## Introduction

Around the world, colorectal cancer (also known as CRC) is one of the leading causes of death and illness. For men, colorectal cancer ranks as the fourth most prevalent kind of cancer, while for women, it is the third most common form of cancer [1]. There are roughly 142 thousand new diagnoses and fifty thousand fatalities that are recorded each year as a result of the illness in the United States [2]. CRC is the second most prevalent newly diagnosed cancer in Australia, with over 14,000 new cases recorded each year. It is also responsible for the second largest number of fatalities attributable to cancer, accounting for the second highest number of deaths after lung cancer. The disease burden is comparable in Australia [3]. As a lifetime risk, CRC is one in seventeen for males and one in twenty-six for women. Direct expenditures associated with colorectal cancer amount to $235 million annually for the Australian government, which accounts for 8.1% of the overall cost of cancer [4]. Both genetic predisposition and environmental variables are responsible for determining the relative risk of CRC, with age being the most significant risk factor for sporadic CRC. The chance of acquiring CRC rises with age, and persons over the age of 50 account for more than 90 percent of all sporadic cases of CRC [5]. Having a family history of colorectal cancer, eating a diet that is low in fibers and folate but rich in fat and red meat, drinking alcohol, smoking cigarettes, working in a sedentary employment, being overweight, and having diabetes are all additional risk factors [6]. Inherited genetic alterations are responsible for around five percent of all cases of colorectal cancer. Some twenty percent of the remaining ninety-five percent of patients have a positive family history, but they are not able to be classified as having any hereditary colorectal cancer condition [7]. It is likely that these are the result of genetic changes that are the result of a hereditary susceptibility, as well as common dietary and environmental influences. A high level of precision may be achieved in the genotyping of hundreds of thousands of single nucleotide polymorphisms (SNP) thanks to recent developments in microarray technology. The goal of genome-wide association studies (GWAS), which make use of this technique, is to identify susceptibility loci for colorectal cancer. In a family-based or case–control design, genome-wide association studies (GWAS) compare the frequencies of genetic variations between afflicted people (cases) and unaffected persons (controls) in order to determine the effects of the disease [8, 9]. Although several susceptibility loci have been found, the utility of these loci in predicting the risk of CRC remains poor [10]. As additional variations are identified, it is expected that the predictive value will improve. Colorectal cancer develops by a series of inherited and environmental alterations that, when combined, cause the normally benign colonic mucosa to become cancerous and invasive. The majority of colorectal cancers originate within pre-existing adenomas, which have some of the genetic fingerprints of pre-cancerous lesions. It is thought that this change would take ten to fifteen years, which will provide doctors with a window of opportunity to screen for and, if necessary, remove premalignant or early malignant tumors. Depending on the characteristics of the polyp, the time it takes for the polyp to advance might vary. Some of the characteristics that are associated with a high risk of quick malignant transformation are a big size (at least one centimeter in diameter), many adenomas (at least three), adenomas with villous alteration, and adenomas with high grade dysplasia [11]. Traditional adenomas are known to exhibit unique molecular and pathological modifications, but the sessile serrated adenomas (SSA) that have been recently characterized exhibit these changes without exception. A separate mechanism, known as the serrated neoplasia pathway, is hypothesized to be responsible for the progression of these lesions into cancer [12]. It has not yet been identified which surveillance technique is the most effective for patients who have SSA, and this process will require further research. In the recent years, the aberrant activation of molecular pathways has been shown to participate in the progression of CRC. The genetic and epigenetic factors have been shown to be dysregulated in CRC [13–18]. Epigenetic factors play a crucial role in the pathophysiology of CRC by regulating gene expression patterns that control cell proliferation, differentiation, and apoptosis. Key epigenetic modifications, including DNA methylation and histone modifications, exhibit aberrant patterns in CRC[19]. These modifications can silence tumor suppressor genes or activate oncogenes, thereby promoting tumor development. Additionally, epigenetic factors contribute to regulating immune responses and inflammatory processes within the tumor microenvironment, influencing immune evasion by tumor cells and anti-tumor immune responses[20]. Clinically, specific epigenetic modification patterns have been associated with CRC sensitivity to chemotherapy and targeted therapies, potentially predicting tumor responses to specific treatment strategies. A comprehensive understanding of the role of epigenetic factors in CRC not only elucidates its pathogenic mechanisms but also provides a theoretical basis for developing new therapeutic targets and personalized treatment strategies [21]. Future research should continue to explore the specific roles of these modifications and their clinical applications to improve treatment outcomes and quality of life for CRC patients. Moreover, the cell death mechanisms including apoptosis [22], autophagy [23], ferroptosis [24] and necroptosis [25] also dysregulate in CRC. These subjects are comprehensively discussed in the current paper.

In the United States, colorectal cancer is the second most prevalent cause of death due to cancer, with approximately 130 thousand new cases and 55 thousand deaths of colorectal cancer each year [26–28]. It is the fourth most common kind of cancer that occurs as an incident. There are a number of environmental risk factors that are shared by colon and rectal cancers, and both types of cancer are seen in people who have distinct genetic abnormalities. However, there are some variances in the root causes of both diseases. During the year 1996, there were around 875 thousand instances of colorectal cancer that were reported all over the world, which accounted for 8.5% of all new cases of cancer [28]. It is estimated that the incidence rates range around 20-fold across the globe, with the industrialized world having the highest rates and India having the lowest rates [29, 30]. The only type of cancer that occurs with almost similar frequency in men and women is colon cancer [31]; however, currently, men have rates that are up to 20% higher than women in areas with high incidence rates, like North America, Australia, Japan, and Italy, where rates are on the rise. The incidence of rectal cancer in males is up to two times higher than that of women. After receiving a diagnosis of colon cancer, the relative survival rate for the next five years is around 55% in the United States [32]. The overall survival rate for patients with rectal cancer may be higher in areas where screening is more prevalent. Both the worldwide disparities and the migrant statistics, as well as the recent quick changes in incidence rates in Italy, Japan, urban China, and male Polynesians in Hawaii [29, 30], demonstrate that colon cancer is particularly susceptible to changes in the environment. It is very uncommon for the incidence rates of immigrants and their descendants to quickly catch up to those of the host nation, and this can even happen within the same generation that migrated [33, 34]. Changing dietary habits and other environmental factors likely account for most of the 20-fold difference between the two nations. Interestingly, while Japan’s incidence rates were low until recently, the world's highest rates are presently recorded among Japanese people living in Hawaii [29]. On the other hand, it has been known for a long time that certain families have a higher incidence of colorectal cancer [35], and there are a number of uncommon genetic disorders that carry a significantly increased risk [36, 37]. Therefore, there is a causal connection between colorectal cancer and both genes and the environment.

## Risk factors of colorectal cancer

Between four and five percent of people throughout the world are estimated to be affected by colorectal cancer. In addition, a great number of personal characteristics or routines are regarded as risk factors since they raise the likelihood of developing polyps or colon cancer [38]. The most important factor that increases the likelihood of getting colorectal cancer is age. After the fifth decade of life, the risk of developing colorectal cancer is significantly raised, although the development of colorectal cancer before the age of fifty is extremely uncommon (with the exception of malignancies that are hereditary) [39]. Not only does one’s age constitute an inherent risk factor, but there are additional risk variables as well. People with a family history of colorectal cancer or inflammatory bowel disease (IBD) are 3.7% more likely to develop ulcerative colitis [40], and people who suffer from Crohn’s disease have a 2.5% higher risk of developing colorectal cancer [41] are also significant risk factors for the development of colorectal cancer. Irritable bowel disease (IBD) is characterized by persistent inflammation, which frequently results in dysplasia, an abnormal cell development. While dysplastic cells may not be malignant just yet, they are more likely to become anaplastic and grow into a tumor if left unchecked. The existence of a positive family history of colorectal cancer in relatives is another risk factor that can be included in this group. This is especially true for relatives who were younger than fifty years old at the time of diagnosis. It is possible for hereditary mutations or environmental factors to be the cause of an elevated risk that is transmitted via a family history [42]. It is possible to lessen the impact of some additional risk factors that are associated with lifestyle by making some little adjustments to one's routines, particularly with regard to one’s eating and physical activity patterns. For example, it is believed that leading a sedentary lifestyle might raise the chance of getting colorectal cancer; however, we still don’t know much about the specific link between sedentary lifestyles and colorectal cancer. Moderate exercise, on the other hand, increases metabolic rate and gastrointestinal motility, and eventually improves metabolic efficiency and decreases blood pressure [43]. Furthermore, environmental factors such as diet, lifestyle, and microbiota also play crucial roles in the development and progression of this disease. Firstly, dietary factors constitute an integral part of colorectal cancer development. Studies indicate that a high-fiber diet and low red meat intake can reduce cancer risk, whereas excessive consumption of processed meat products may increase it. Additionally, adequate intake of vitamins and minerals is closely associated with colorectal cancer development [44]. Secondly, lifestyle factors are equally important in the development of colorectal cancer. Regular physical exercise, weight management, and smoking cessation are closely linked to reducing the risk of colorectal cancer. These findings highlight the potential for preventing colorectal cancer through lifestyle modifications. Moreover, the balance of gut microbiota also significantly influences the development of colorectal cancer [45]. Research suggests that specific microbial communities in the intestines can alter host metabolism and immune responses, thereby affecting tumor formation and progression. In summary, while genetic factors play a crucial role in colorectal cancer, environmental factors are equally pivotal [46]. Future research should delve deeper into exploring the specific connections between these environmental factors and colorectal cancer to develop more effective prevention and treatment strategies.

Another major risk factor for colorectal cancer is obesity, which is associated with a sedentary lifestyle. Consumption of food and the development of visceral adipose tissue (VAT), a hormonally active subset of total body fat, are surprisingly linked to this increased risk. Insulin resistance, alterations to metabolic enzymes such as adiponectin or lectin, and the release of proinflammatory cytokines all contribute to an inflammatory state in the colon and rectum, which in turn increases the risk of colorectal cancer [47]. In this setting, food plays a major role in the risk of colorectal cancer; in fact, poor dietary habits can increase the risk of colorectal cancer by 70% [48]. Intestinal heme group release from red meat, for instance, increases the production of cytotoxic and genotoxic aldehydes and carcinogenic N-nitroso compounds via lipoperoxidation [49]. Furthermore, after digestion, meat that has been cooked at high temperatures produces heterocyclic amines and polycyclic hydrocarbons. These compounds are thought to have carcinogenic potential [50]. There is mounting evidence that cigarette smoking and alcohol consumption both raise the risk of colorectal cancer. One of the main byproducts of alcohol consumption, acetaldehyde, has been identified as a carcinogen. This is due to the fact that in populations where polymorphisms of alcohol metabolism enzymes play a significant role, it raises the risk of colorectal cancer [51]. However, the exact nature of the link between alcohol use and colorectal cancer remains unclear. Contrarily, research has demonstrated that cigarette usage can increase the incidence of colorectal cancer by up to 10.8 percent [52]. This is because nicotine and other carcinogens found in tobacco have metabolites that can swiftly reach the intestines and form polyps [52, 53]. Although there is some evidence that smoking increases the risk of colorectal cancer (CRC), the strongest link has only been seen in those who have smoked for many years, regardless of whether they have ever tried to quit [54].

## Molecular pathways and genomic changes in colorectal cancer

### Wnt pathway

Most research and characterization efforts have focused on CIN, one of numerous colorectal routes [55]. The tumorigenic process involves several proteins and regulators of mitotic spindle checkpoints [56, 57]. When it comes to mitotic chromosomal integrity, several proteins and regulators play a role. There is consensus that the adenomatous polyposis coli (APC) tumor suppressor gene’s early mutation was a “key” one. Both sporadic CIN and familial adenomatous polyposis (FAP) are associated with this gene when it is germline mutated [58, 59]. There is a germline mutation of the APC gene that has been detected in sixty percent to eighty percent of families that have FAP syndrome [60]. The colorectum develops hundreds to thousands of adenomas during adolescence and young adulthood in patients with FAP syndrome, an autosomal-dominant hereditary disease. A variation of FAP called attenuated FAP (AFAP) is defined by having less than 100 adenomas. Germline mutations in the APC gene, affecting either the 5' or 3' region, create this FAP variant. Interestingly, a syndrome called MUTYH-associated polyposis (MAP) is seen in 16% to 40% of patients with less than 100 polyps. This condition is marked by the bi-allelic inactivation of the MUTYH related excision repair gene. There is a lot of overlap between the phenotypes of AFAP and MAP [61]. The APC tumor suppressor gene is involved in the APC/β-catenin/Tcf pathway. Because it stops the degradation of β-catenin, this protein’s inactivation causes the WNT pathway signaling to rise. The increase in the proliferation, differentiation, migration, and adhesion of colorectal cells is caused by the activation of the TCF-targets, which are brought about by the translocation of β-catenin from the cytoplasm into the nucleus, caused by the concentration of β-catenin in the cytoplasm. In the early phases of colorectal pathogenesis, mutations in CTNNB1 are present and may replace mutations in APC in the initiation stages [62, 63]. In colorectal cancer and spontaneous colorectal cancer, these mutations can be discovered in genes that are involved in the APC/β-catenin/Tcf pathway, even in patients without APC mutations. Specifically, tumors lacking APC mutations are detected in 48% of cases with β-catenin mutations [62]. Furthermore, different parts of the WNT/APC/β-cat pathway can be altered either directly or indirectly. For example, this can be accomplished by constitutively activating β-catenin or Tcf. It was found that the mitotic checkpoint protein BubR1 is one of the regulatory genes that interact with the APC suppressor gene. That mechanism is crucial. In addition to Bub1, Bub3, Mad1, Mad2, Mad3, Mps-1, and CENP-E, BubR1 is a part of the machinery that controls mitochondrial checkpoints. It binds to Cdc20, which inhibits APC activity and stimulates a “wait anaphase” signal [64]. The fact that its downregulation and subsequent inactivation cause polyploid cell formation, prolonged cell survival, and excessive proliferation suggests a potential pathogenic mechanism in the onset of chromosomal instability in sporadic types of colorectal cancer. While oncogenes regulate β-catenin activity at various levels, mutations in these genes can indirectly increase its activity. Many components of the Notch pathway, which are crucial regulators of cellular differentiation and have now been found to play a role in the development of colorectal cancer, interact reciprocally with the protein β-Catenin [65]. The results shown by Kwon and colleagues indicate that Notch1 enhances the buildup of active β-Catenin protein, and this enhancement happens independently of ligand-receptor activation [66]. Furthermore, it was found that the Notch pathway activity is dose-dependently reduced with long-term usage of non-steroidal anti-inflammatory drugs (NSAIDs), mostly ibuprofen. Extensive study has shown that nonsteroidal anti-inflammatory medicines (NSAIDs) can reduce the risk of colorectal cancer. This supports those findings [66]. In addition to the amplification of the CDK8 (cyclin dependent kinase-8) gene, which is situated at 13q12.13 and is present in around sixty percent of cases of colorectal cancer, there are other genetic perturbations that have the ability to alter the activity of β-Catenin. Within the context of colorectal cancer, an elevated level of CDK8 kinase activity functions as an oncogene by promoting the expression of β-Catenin [67] and Notch1, hence enhancing transcription and promoting cell differentiation [68]. Findings from previous studies are consistent with those of Firestein et al., who found a strong relationship between CDK8 expression and β-catenin activation, overexpression of fatty acid synthase (FASN), and p53 expression. Colorectal cancer patients with CDK8 over-expression also had a significantly worse prognosis [69]. Recent research has demonstrated that activation of the G-protein-coupled orphan receptors LGR-4 and LGR-5 can enhance signaling through interactions with R-respondin family members. The WNT signaling pathway is known to be amplified by these proteins. Their findings indicate that the researchers were able to increase the activity of Wnt/β-Catenin by improving the phosphorylation of the WNT co-receptor LRP6 [70]. Furthermore, it was found that Cyclin D1 (CCND1) is involved in APC signaling. As with other cyclin-dependent kinases like p27 (CDKN1B) and p21 (CDKN1A), CCND1 is essential for cell cycle regulation, but it is most significant as the cell moves from the G1 to the S phase [71]. Colonic neoplasia develops in part because of the cell's ability to escape apoptosis, which is facilitated by the overactivation of CCND1 due to an APC mutation. The frequency of CCND1 in cancer, normal colonic mucosa, and normal colonic mucosa was studied by Arber and colleagues. The researchers discovered that CCND1 expression was significantly higher in colorectal cancer patients' mucosa [72]. Finally, in their study on preventing colorectal cancer in obese people, Morikawa and colleagues found that being overweight and not getting enough exercise both raise the risk of acquiring the disease, but none of these factors affects the WNT/beta-catenin pathway [73].

### Estrogen pathway

An active ER can stimulate gene transcription by either directly interacting with certain DNA sequences that are referred to as estrogen response elements (ERE) or by interacting with other transcription factors such as c-Jun and/or c-Fos, which ultimately results in transcription [74]. Although the relationship between ERα and other transcription factors is less extensive, it does involve c-Jun and c-Fos of the activating protein-1 complex (AP1) and SP1 [75]. This is so even though there is little difference in the way the two ERs interact with ERE. By studying AP1, scientists have shown that E2 binding to ERα activates transcription, but E2 binding to ERβ decreases transcription by rerouting estrogen from the ERα pathway [76, 77]. Additionally, estrogen and its receptors have the ability to activate several signaling pathways without having to directly contact with DNA. This can result in the modification of various cellular processes, in addition to the impact that they have on the genome. It has been demonstrated that transmembrane ERs are capable of activating a wide variety of intracellular pathways, such as protein kinase C (PKC) [78], intracellular Ca2 +  [79, 80], cytosolic cAMP [81], nitric oxide [82], and MAPK [83]. Transmembrane ERα signaling, which is mediated by PI3K, has the potential to contribute to the proliferation and survival of cells. On the other hand, transmembrane ERβ signaling leads to the accumulation of Ca2 + within the cell, which ultimately leads to the inhibition of PKC signaling. One additional function of ERβ is to modulate the regulation of the cell cycle by interacting with c-Myc, cyclin D1 [84], and cyclin A [85], which ultimately leads to the suppression of the advancement of the cell cycle. However, the levels of ERα expression remain low in both normal colonocytes and malignant colonocytes. ERβ, on the other hand, is the most prevalent ER in the normal colon [86, 87], and its expression level is greater in the ascending colon [88]. The amount of expression of ERβ in tumor tissue is lower when compared to the normal mucosa of the colon, and this drop is correlated with the stage of the illness [89, 90]. Through their research, Hartman and his colleagues demonstrated that the introduction of ERβ into SW480 cell lines led to the suppression of proliferation and the arrest of the cell cycle. The weight of the tumor was reduced by 70% in SW480 xenografts that expressed ERβ, as reported in reference [91]. In addition, a notable increase in the number of polyps was observed in ApcMin/ + mice after ERβ deletion, and the addition of E2 treatment failed to halt the development of polyps in these mice [92]. Through their research, Edvardsson and his colleagues demonstrated that the MAPK signaling pathway is influenced by the transfection of colon cancer cell lines with ERβ [93]. In addition, the expression of ERβ leads to the downregulation of interleukin-6, which ultimately leads to a reduction in inflammation [93]. According to the hypothesis put forward by Giroux and colleagues, the effects of ERβ in ApcMin/ + mice are attributed to the regulation of the TGFβ signaling pathway [92]. A number of studies provide evidence that Wnt/β-catenin signaling and ERα engage in cross-talk with one another. Within the context of a particular experiment, the activation of Wnt signaling was achieved by the transfection of SW480 and HCT116 with ERα. However, the addition of an ER antagonist led to the deactivation of the pathway. In addition,using an antibody against β-catenin activated ERE in a manner dependent on estrogen, as demonstrated by this experiment's execution [94].

### The microsatellite instability (MSI) pathway

The presence of MSI is yet another significant kind of genomic instability [95]. The term “microsatellites” refers to nucleotide repeat sequences that are dispersed throughout the genome. The term “microsatellite instability” (MSI) describes a disparity, and consequently instability, in the amount of nucleotide repeats that are present in microsatellite areas of tumor DNA as opposed to germline DNA. During the process of duplicating these short repetitive sequences, DNA polymerase is more prone to make mistakes, and as a result, mismatch repair (MMR) malfunction happens, which leads to mismatch instability (MSI). There are at least seven proteins that make up the MMR system. These proteins are mlh1, MLH3, msh2, msh3, msh6, pms1, and pms2. These proteins create functional heterodimers by forming associations with these particular partners.There are five functional heterodimeric proteins that are formed by MLH1 and MSH2, which are key components of the mismatch repair machinery [96]. This set of proteins is known as MSH2-MSH6, MSH2-MSH3, MLH1-PMS1, MLH1-PMS2, and MLH1-MLH2. Mutations in several genes, including MLH1, MSH2, MSH6, and PMS2, have been associated with the existence of HNPCC. But most colorectal tumors with a functional MMR system will only contain frameshift mutations at a small handful of microsatellites. Therefore, to guarantee that researchers and medical professionals were using the same definitions, a standardized panel of microsatellites was created [97]. Currently, the panel is being supported by three dinucleotide microsatellites (D5S346, D2S123, and D17S250) and two mononucleotide microsatellites (BAT25 and BAT26). The presence of MSI at least two (40%) of the five necessary locations is considered a major MSI, also known as MSI-high (MSI-H). When microsatellite instability is present at a single site, we say that the MSI is low (MSI-l), and when there is no instability at these markers, we say that the MSS is present. MSI is responsible for a significant rise in the number of genetic mistakes, and a number of microsatellites are found in genes that are associated with the development of colorectal cancer. These genes include MSH3, TGFBR2, BAX, CASP5, MSH6, CTNNB1, APC, IGF2, and E2F4 [98]. The reason why most MSI-H cancers are diploid is because MMR failure causes genomic instability without the physiologic need for a corresponding CIN. In rare cases, most MSI-H CRCs develop when the MLH1 promoter is DNA methylated, which suppresses MLH1 expression through transcription. This is in contrast to the pure form of MSI, which is caused by HNPCC. The CIMP pathway is believed to be a component of these malignancies since they display both CIMP and MSI, as described in this article. However, the biology of MSI-H tumors is same regardless of whether they are hereditary or spontaneous.

### Chromosomal instability

The vast majority of cancerous disorders are characterized by some kind of genetic instability [99, 100]. There is a high incidence of chromosomal insertions, inversions, deletions, and rearrangements (also known as CINs) in sporadic colorectal cancers [101]. These genetic modifications include insertions, inversions, deletions, and rearrangements. In clonal expansion, chromosomal instability (CIN) is a phenomenon that occurs when the chromosomal content of cells changes at a pace that is greater than the typical rate [102]. On the other hand, the criteria for CIN are not as well defined as those for microsatellite instability (MSI). CIN, on the other hand, causes a change in the pattern of gene expression. This change can be the result of insertions or deletions that modify the dosage of the gene, or it can be the consequence of structural modifications such as rearrangements that have the potential to be responsible for a gene being regulated by another promoter. The measurement of changes in chromosomal content from one cell generation to the next is rather challenging due to the requirement of specialized technology that measures the variability from cell to cell as well as the increasing rate of instability occurring in cells. Flow cytometry, fluorescent in situ hybridization, and comparative genome hybridization are some of the methods that are widely utilized for the purpose of determining the copy number status of a tumor. The acronym CIN is frequently used to refer to the detection of extensive chromosomal abnormalities. The presence of aneuploidy or a complicated karyotype is not synonymous with CIN, despite the fact that CIN frequently leads to aneuploidy. Through the course of history, tumors that exhibited MSI have been considered to be diploid, whereas tumors that did not exhibit MSI have been referred to be CIN [101]. This narrow categorization, on the other hand, is beginning to lose its relevance as tumors that exhibit both or none of the characteristics have been reported [103]. At this time, the precise mechanism that causes CIN has not been uncovered. Genes that have been functionally categorized as cell cycle checkpoint, mitotic spindle, chromosomal segregation and condensation, and sister chromatid cohesion genes are among the many genes that have been postulated to be responsible for CIN [104]. An analysis of chromosomal unstable tumors found that mutations in APC, KRAS, SMAD4, and TP53 are statistically significantly more common [102]. On the other hand, cytogenetic investigations have demonstrated that gains and losses of chromosomal material are confined to certain chromosomes [105, 106]. Carcinomas of the colon have complicated karyotypes. Despite the fact that genes associated with cancer are found in a number of these areas, neither of these abnormalities has been identified as either a cause or a consequence of CIN. With the exception of chromosomal 8 and 16 rearrangements, which have been found to be associated with clinical outcome [107], very few of the chromosomal alterations that have been discovered have clinical consequences for patients who have been diagnosed with colorectal cancer as of at this time.

### EGFR/MAPK axis

One type of transmembrane protein with an extracellular ligand-binding domain is a catalytic receptor tyrosine kinase (RTK). Among the RTK family, EGFR is an essential member [108]. Autophosphorylation of many intercellular domain tyrosine residues occurs after ligand contact, which triggers EGFR activation and dimerization. Furthermore, the rat sarcoma virus (RAS) is activated by the EGFR adaptor protein complex, which binds to phosphorylated tyrosine residues and converts guanosine diphosphate (GDP) to guanosine triphosphate (GTP). Grb2 and SOS are the components of this complex. There is a cascade of kinases that are activated through phosphorylation when the receptor-activated protein kinase (RAS) is engaged. These include MAPK, ERK, MAPKK, and mitogen-activated protein kinase kinase-melphalan (MAPKK) [109]. The ERK signaling pathway controls cell survival, differentiation, and proliferation, according to multiple studies. Several human cancers have EGFR/MAPK signaling pathway dysregulation. The fact that it can promote tumor growth and malignant transformation via increased cell proliferation, prolonged survival, angiogenesis, anti-apoptosis, invasion, and metastasis is a major reason for this [110]. The EGFR/MAPK signaling pathway has been discovered to be directly connected to the oncogenic processes that are associated with CRC, and it has been found to play key roles in the formation of CRC tumors and the progression of the disease [111]. Because of this, this system and the downstream signaling cascades that it is associated with have been identified as potential therapeutic intervention targets for colorectal cancer [112, 113].

### PI3K *axis*

An important intracellular lipid kinase, PI3K regulates several cellular activities, such as proliferation, migration, differentiation, and survival [114]. The PI3K molecule is structurally heterodimeric, with the regulatory subunit p85 and the catalytic subunit p110 making up its two halves. The processes by which PI3K affects tumor formation and progression are regulated by protein kinase B (AKT/PKB), a serine/threonine-protein kinase (Ser/Thr kinase) and a downstream effector of PI3K [115]. The phosphorylation of AKT was shown to have a role in the prevention of apoptosis and the proliferation of cells derived from human colorectal cancer. Because of this, the suppression of the PI3K/Akt pathway was utilized as a therapeutic for a variety of malignancies [116]. The binding of ligands to receptor tyrosine kinases (RTK) is what causes PI3K to become active. In the subsequent step, phosphorylation of phosphatidylinositol 4,5-bisphosphate (PIP2) by active PI3K results in the formation of phosphatidylinositol 3,4,5-trisphosphate (PIP3). In the following step, PIP3 activates AKT by binding to its serine and threonine residues, which ultimately leads to the survival and proliferation of cells. By regulating downstream proteins like the mammalian target of rapamycin (mTOR), which is responsible for mediating the cell cycle, proliferation, angiogenesis, protein translation, as well as growth and survival, AKT is able to control the activity of these proteins. PIP3 is dephosphorylated by a protein called phosphatase and tensin homolog (PTEN), which is recognized as a tumor suppressor and a PI3K pathway downregulatory protein. This abnormal expression of the pathway is frequently seen in colorectal cancer, which leads to the unending expansion of cells and ultimately ends in the development of cancer. In general, it has been observed that the PI3K signaling pathway has an oncogenic role in the beginning stages of colorectal cancer and its progression. Aberrant Wnt signaling can enhance EGFR signaling, as β-catenin has been shown to increase the expression of EGFR, leading to enhanced MAPK signaling. Additionally, KRAS mutations, commonly found in the EGFR/MAPK pathway, can also affect Wnt signaling dynamics. The activation of EGFR leads to the simultaneous activation of both the MAPK and PI3K/AKT pathways, which can converge at multiple nodes, such as mTOR, influencing cell growth and survival. Furthermore, the PI3K pathway can be activated by Wnt signaling through the inhibition of GSK-3β, a kinase that phosphorylates β-catenin, thus preventing its degradation. This interaction promotes β-catenin stabilization and nuclear accumulation, enhancing Wnt target gene expression.

### TGF-β *axis*

The TGF-β signaling pathway is known to impact several cellular activities, such as proliferation, growth, differentiation, division, migration, and adhesion. Signaling by TGF-β is initiated when its ligand binds to its receptors. Receptor dimerization brings about this effect by bringing the two heterodimer receptors into a complex. The next step involves phosphorylation of the receptors' kinase domain, which activates the transcription factors SMAD proteins located farther downstream. Specifically, SMAD2 and SMAD3 activate each other by forming phosphorylated heterodimers, which bind to SMAD4 to form a heterotrimer. To manage transcription, the heterotrimers move into the nucleus and attach to the genes that are targets of TGF-β. Several recent investigations have shown that TGF-β, in its role as a tumor suppressor, mediates cell division, proliferation, apoptosis, and differentiation in colon epithelial cells [117]. As a result of the loss of TGF-β in colorectal cancer cells from the beginning stages, growth inhibition resistance is frequently found. On the other hand, in the latter stages of colorectal cancer, there is an alteration in the expression of TGF-β, which results in the shift from epithelia to mesenchymal tissue (EMT). The usual cellular immune response was diminished as a consequence of the enhanced invasion and cell migration that occurred as a consequence of this. It is also possible for TGF-β to promote EMT through a mechanism that is independent of SMAD4, namely through the Ras homolog family member A (RhoA) signaling pathway of the cell [118].

### Tyrosine kinase receptor pathway

The receptors of many hormones and polypeptide growth factors are proteins with an intrinsic tyrosine kinase activity and a single transmembrane domain [119]. Biological cells include these receptors. Some examples of such receptors are fibroblast growth factor receptors (FGFR), platelet-derived growth factor receptors (PDGFR), vascular endothelial growth factor receptors (VEGFR), and epidermal growth factor receptors (EGFR). A member of the dimeric receptor family, the insulin-like growth factor receptor (IGFR) is another type of tyrosine kinase receptor. The human epidermal growth factor receptor (HER) is one of four proteins that are known as the ErbB/HER receptors. It is closely related to the v-ErbB oncogene of the avian erythroblastosis virus, which causes erythroid leukemia in birds. Since malignancies are linked to overexpression of the human ErbB2 gene—which codes for the human EGFR, often known as HER2—id [120], the connection between ErbB2/HER2 and cancer has also been detected in humans. An interaction between a growth factor and a tyrosine kinase receptor's extracellular domain triggers the formation of a dimer. The adjacent receptor is autophosphorylated on many tyrosine residues as a result of this dimerization. The majority of cytoplasmic proteins that are part of the growth factor signaling pathway share domains with the protein SRC, sometimes shortened to “sarc” for “sarcoma.” One of these domains, known as SH2, binds to phosphorylated tyrosine, and the other, SH3, binds to a part of a protein that has a polyproline helix secondary structure. These two domains are known as SH2 and SH3, correspondingly. The ability to convert GDP to GTP in a GTP activating protein (GAP) is possessed by guanine exchange factors (GEFs). Connecting the receptor to a GEF is the function of GRB2, a protein with SH2 and SH3 domains. A key Ras GTPase-activating protein (RasGAP), Son of Sevenless (SOS) is one of several. Ras becomes activated because of this. Three separate Ras genes—H-Ras, N-Ras, and K-Ras—are found in the human genome. On the order of 30% of human cancers involve cells expressing mutant Ras oncogenes. Ras initiates the mitogen-activated protein kinase (MAPK) cascade when it is linked to GTP, which in turn stimulates a family of serine/threonine protein kinases. The initial Ras-activated kinase, RAF-1, is a component of this cascade [121], which is a mitogen-activated protein kinase kinase kinase; MEK, which is an intermediate mitogen-activated protein kinase kinase; and ERK, which is a mitogen-activated protein kinase that phosphorylates many target proteins in both the cytosol and the nucleus. Elk-1 is a transcription factor that stimulates the activation of several genes; ERK phosphorylates it in the nucleus. To phosphorylate the nucleus-resident transcription factor c-Jun, another mitogen-activated protein kinase called JNK (c-Jun N-terminal kinase) is responsible. Ras can activate several cascades, including the RAF-MEK-ERK and MAPKKK-MKK-JNK cascades, the TIAM1-Rac-Rho cascade, the Ral-PLD cascade, and the TIAM1-Rac-Rho and TBK1-NFκB cascades [122]. The PI3K-PDK-AKT-mTOR receptor is one of the tumor-initiating downstream targets of EGFR. Phosphatidylinositol 3-kinase (PI3K) is responsible for phosphorylating PIP2 into PIP3. Phosphate and tensin homologue (PTEN) is a tumor suppressor gene that hyperactivates PI3K signaling to block AKT activation. The PI3K/AKT signaling pathway component PIP3 is dephosphorylated to do this. The final product, called mTOR (mammalian target of rapamycin), damages DNA [123, 124].

### P53 pathway

There are several routes via which the p53 protein, an important transcription factor, inhibits tumor formation and progression [125–127]. Despite its reputation as the “Guardian of the Genome” for its crucial role in maintaining DNA integrity, p53 signaling is often dysregulated in colorectal cancer. Negative regulators of p53, MDM2 and MDM4, normally keep cellular p53 activity in control under homeostatic conditions. The combination of these regulators leads to the proteosomal degradation and ubiquitination of p53, which keeps the tumor suppressor at ineffective quantities in cells. However, following DNA damage, the first step in cellular stress is the transfer of signals from certain stress-sensing proteins to transducer and effector kinases. These kinases then modify p53 by post-translational modification. This mechanism causes a conformational shift, which in turn stops MDM2/MDM4 from binding to p53. This allows p53 to stabilize and become active [128, 129]. In its activated state, the p53 protein can transactivate several genes farther downstream by binding to DNA in a sequence-specific fashion. A prime target is p21, an inhibitor of the cyclin-dependent kinase (CDK) that enables cells to repair DNA damage and survive by preventing the cell cycle from progressing further. Actually, several parts of the DNA repair machinery can be activated by the p53 protein, which can further aid the process [127]. At high enough damage levels, the p53 protein transactivates genes like Bax, Puma, and Noxa that are involved in cell death, and it transrepresses genes like survivin and Pdk2 that are involved in tumor development and anti-apoptosis. This tips the scales in favor of programmed cell death, which stops the transmission of potentially cancer-causing DNA to offspring [128, 130, 131]. However, extremely high levels of gene alterations can hinder the tumor-suppressing action of p53 in colorectal cancer. Because of these mutations, p53’s structural conformation changes, rendering it unable to bind to DNA in a sequence-specific way and rendering the p53 signaling pathway inactive. Furthermore, gain-of-function phenotypes can be brought about by some mutations that allow mutant p53 to bind off-target DNA. This, in turn, speeds up the development of prostate cancer and boosts pro-survival pathways [128]. In colorectal cancer, mutations in the p53 gene appear to take place during the latter stages of the transition from adenoma to carcinoma [132, 133]. A large-scale study with 3583 colorectal cancer patients found that p53 mutations are more common in tumors located farther from the colon or rectal area compared to tumors located closer to the colon itself (45% vs. 34% on average) [134]. The majority of these changes, over 80 percent, are missense mutations, mostly found in exons 4–8. Furthermore, almost fifty percent of these mutations are found in the five hotspot areas, namely at amino acids 175, 245, 248, 273 and 282 [132, 134, 135].

**Fig. 1:**
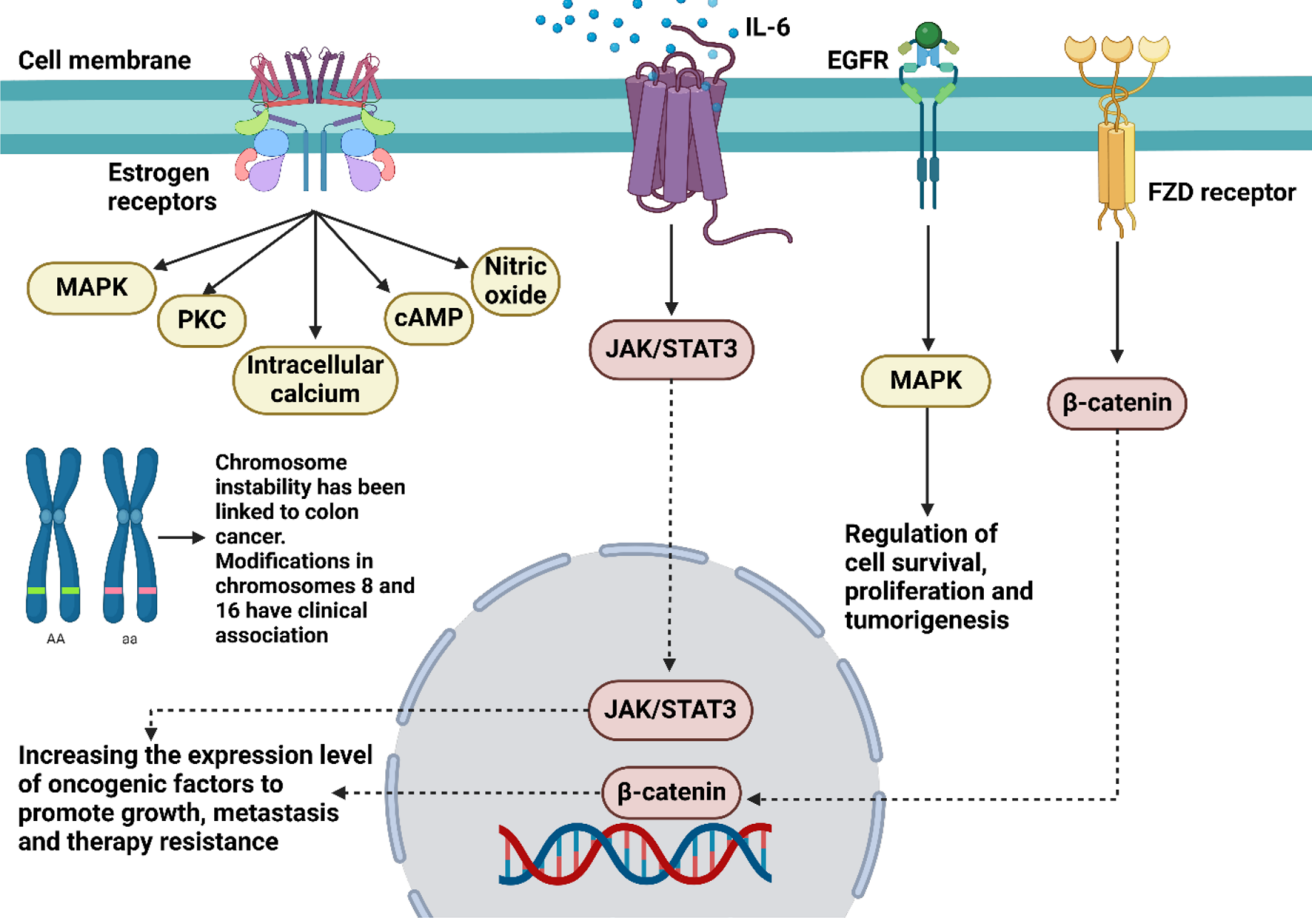
The major pathways dysregulated during the progression of colorectal cancer

Examining the p53 pathway in colorectal cancer via the perspective of medication resistance can provide valuable insights. For this disease, doctors use a wide variety of drugs, including as the small molecules capecitabine, irinotecan (CPT-11), oxaliplatin, and 5-fluorouracil (5-FU) [136]. All of these medications are administered in doublet or triplet combinations. Additionally, the unfavorable impact of mutation on antitumor effects has been shown in colorectal cells in vitro [137, 138] and in patients [135]. It is interesting to note that these medications are dependent on wild-type p53 [139, 140]. Some studies have struggled to use p53 status as a predictor of treatment efficacy because wild-type and mutant colorectal tumor cells do not respond in a clearly differentiated manner. These studies include those involving 5-FU [141] and oxaliplatin [142]. However, this might be because the mutation does not inhibit drug-induced activation of p53 function, as seen in HCA7 colorectal cells, or because wild-type p53 in some tumors does not become activated after being exposed to therapeutic drugs, as shown in the NCI-747 colorectal tumor model [143]. Therefore, for around half of the 300 uncommon (non-hotspot) mutations discovered in multiple types of human cancer, mutant p53 may continue to play a significant role [144]. Two high-quality studies analyzed 1180 p53 mutants from 3583 colorectal cancer clinical cases. The results showed that 36.2% (105/290) of the mutant p53 in proximal colon cancer, 37.3% (44/118) in distal colon cancer, and 27.5% (212/772) in rectal cancer retained significant activity (Fig. 1) [134, 145].

## Cell death in colorectal *cancer*

### Autophagy

Cells gather proteins and organelles by a process known as autophagy, which is a mechanism that has been preserved throughout evolution [146]. This process then transports the cargo to the lysosomal compartment, where it is ultimately destroyed and recycled [147]. It is becoming increasingly clear that autophagy has consequences not only for the physiology of cells but also for the beginning and progression of a wide range of illnesses, including cancer [148, 149]. When there is a disturbance in the flow of autophagy, there is a buildup of organelles, protein aggregates, and lipid droplets within the cell. There is a possibility that these accumulations may result in the generation of reactive oxygen species and will give rise to metabolic deficiencies. A disturbance of autophagic flow can promote carcinogenesis, particularly in situations that are stressful and in environments where there is a lack of energy. Specifically, the allelic deletion of the crucial autophagy protein Beclin 1 (also referred to as Atg6) is responsible for the development of head and neck cancer in mice [150, 151]. On the other hand, autophagy is very necessary for the survival of cancer cells, and cancer cells exhibit an exceptionally high amount of autophagy. On the other hand, the activation of autophagy is beneficial to survival in cases when growth factor withdrawal and hypoxia are present [152]. The production of autophagosomes is especially noticeable in tumors that are developing in an environment that is low in oxygen. Taking into consideration these findings, anticancer medicines that suppress autophagy are a promising potential treatment. A number of clinical trials are presently being conducted to investigate the effectiveness of the anti-malaria medication chloroquine, which is known to block autophagy (www.clinicaltrials.gov) [153]. Preclinical research has been conducted on a number of additional substances or medications that are known to regulate autophagy and have been examined as potential therapy options for colorectal cancer [154–157]. It has been demonstrated that chloroquine is efficient in reducing 5-FU resistance in colorectal cancer cells in vitro [158, 159]. Cetuximab, a chimeric anti-EGFR antibody that has been authorized for use, is able to exert its anticancer impact at least partially through autophagy-induced cell death [159].

Despite the fact that it may seem counterintuitive, medicines that directly induce autophagy are also being investigated for use as therapeutic methods in colorectal cancer. The mammalian target of rapamycin is a significant target that can activate autophagy in colorectal cancer cells, which can ultimately lead to death [160]. A relatively new drug called Everolimus, which is derived from rapamycin, has just been developed for the treatment of colorectal neuroendocrine tumors [161]. In a Phase II research, Everolimus demonstrated adequate tolerability; however, it was not able to demonstrate substantial benefit in patients who had received extensive pretreatment for metastatic colorectal cancer [162]. According to the findings of another clinical trial, the combination of tivozanib, an inhibitor of vascular endothelial growth factor receptor tyrosine kinase, and everolimus led to stable disease in fifty percent of all patients with metastatic cancer who participated in the study [163, 164]. The significant role that autophagy plays in the development of colorectal cancer is brought to light by these findings, which are partially conflicting. There is a significant overlap between the signaling networks for apoptosis and autophagy, which is an important fact. First and foremost, Bcl-2 proteins have the ability to block both apoptosis and autophagy by their binding to the proautophagic Beclin1 protein pathway. Accordingly, it has been demonstrated that BH3-mimetics are capable of inducing both apoptosis and autophagy. Through the facilitation of autophagy and apoptosis, for example, ABT-737 has the ability to cause cell death in colorectal cancer cells in a manner that is synergistic with the COX2 inhibitor celecoxib [165, 166].

Cell death pathways play a crucial role in the development and treatment of CRC, with their molecular mechanisms and variations across different subtypes or stages influencing tumor growth, treatment responses, and patient prognosis [167, 168]. Firstly, apoptosis, as the principal programmed cell death pathway, exhibits intricate regulatory mechanisms in CRC. Apoptosis is typically executed through the activation of caspase enzymes, either via the mitochondrial pathway or death receptor pathway. Different CRC subtypes may demonstrate varying sensitivities and responses to these pathways. For instance, microsatellite instability-high CRC (MSI-H) often displays mitochondrial dysfunction or abnormal expression of Bcl-2 family proteins, potentially leading to alterations in apoptosis pathways and the development of drug resistance [167, 169]. Secondly, necrosis, as a non-programmed form of cell death, manifests differently across various stages of CRC. Necrosis typically involves cell membrane rupture and leakage of cellular contents, processes that can influence tumor growth and dissemination through inflammatory reactions and immune cell involvement. Lastly, autophagy, a critical mechanism for maintaining cellular homeostasis, also plays a significant role in CRC development. Autophagy degrades harmful proteins and damaged organelles via lysosomes, influencing cell survival and metabolism [170, 171]. Different CRC subtypes or treatment stages may exhibit varying levels of autophagic activity and regulatory mechanisms, directly impacting tumor sensitivity to drug therapies and patient prognosis. Overall, a comprehensive understanding of the specific molecular mechanisms of cell death pathways in CRC and their variations across different subtypes or stages helps elucidate the complex biological characteristics of this disease and the potential for personalized therapies. Future research should further explore the dynamic changes in these pathways and identify novel therapeutic targets to enhance treatment efficacy and patient survival rates.

### Apoptosis

In order to absorb a wide variety of cellular strains into a variety of responses, including apoptosis, the tumor suppressor gene known as P53 is responsible for this transformation [172]. The product of this enzyme binds to certain regions in DNA and regulates the transcription of a number of pro-apoptotic genes, including Bax and the BH3-only proteins puma and noxa [173]. In addition to causing the release of cytochrome c from mitochondria, these genes and proteins are responsible for the inactivation of the anti-apoptotic proteins Bcl-2 and Bcl-xL. Furthermore, P53 is responsible for the promotion of the production of apoptotic effector proteins such APAF-1 and caspase 6 [174]. P53 also houses the death receptor Fas and DR5, two main players in the extrinsic apoptotic pathway; additionally, it contains the BH3-only protein Bid, which connects the extrinsic system to the intrinsic pathway[175]. In order to prevent caspase activation, P53 suppresses the primary inhibitor of apoptosis proteins (IAP) gene. But this gene still hasn’t had its full potential investigated. Additionally, P53 blocks survival pathways that neutralize apoptosis, such as the PI3 kinase/AKT survival pathway. One way this is achieved is by increasing the transcription of PTEN, which is a PI3 kinase inhibitor, phosphatase and tensin homolog. This, in turn, prevents MDM2 from inhibiting P53 [176]. Additionally, in addition to apoptosis, P53 is capable of committing to the detention of the cell cycle, the mending of DNA, and senescence. It is also possible for the P53 protein to react to DNA damage by either causing a growth arrest during the G1 or G2 phase of the cell cycle or by causing the cell to die automatically. In a similar manner, P53 protects the cells against the replication of damaged DNA on a regular basis [174]. Consequently, the deletion of the P53 gene through the CIN pathway is an excessively strong factor in the progression of adenoma to carcinoma in colorectal cancer [177]. Additionally, it has been demonstrated that P53 has a significant impact on the patient's responsiveness to the chemotherapeutic drugs that are employed in the treatment of colorectal cancer [178]. P53 gene deletions and mutations have been observed in as much as 85 percent of colorectal cancers, according to reports. These mutations and deletions often take place during the transition from adenoma to adenocarcinoma [135]. It is advantageous for cells to have a malfunctioning P53 because these cells are able to tolerate chromosomal instability caused by telomere limitation and have an influential selection advantage. A number of instances in which the failure of apoptosis plays a crucial role in the evolution of malignant clones include the adenoma/carcinoma transition that occurs in CRC. A small number of immune-histochemical investigations, on the other hand, did not support the primary role of mutant P53 protein as an inhibitor of apoptosis in the development of colorectal cancer [179].

### Immunogenic cell death and drug resistance

When it comes to adjuvant and metastatic colorectal cancer treatment, cytotoxic chemotherapy is still the gold standard [180]. The fundamental component of the majority of regimens is 5-FU, a pyrimidine analogue that blocks thymidylate synthase. It is common practice to combine 5-FU with oxaliplatin or irinotecan, two topoisomerase I inhibitors or fluorouracil, leucovorin, oxaliplatin, or fluorouracil, leucovorin, oxaliplatin, irinotecan, all in the acronym FOLFOX [181, 182]. Typically, targeted biologic medicines like cetuximab or panitumumab, which are monoclonal antibodies against epidermal growth factor receptor (EGFR) or anti-angiogenic (bevacizumab), are also added to the chemotherapy backbone for increased efficacy [181]. There has been a lack of research on bevacizumab's capacity to cause ICD. On the other hand, a mouse model has demonstrated that cetuximab, either alone or in conjunction with FOLFIRI, can produce ICD [183]. The relationship between immunogenic cell death (ICD) and drug resistance is multifaceted and primarily manifests in the following ways: firstly, certain anticancer drugs (such as anthracyclines and some platinum compounds) can induce ICD, enhancing the immune system's ability to recognize and eliminate tumor cells, thereby increasing the drug's efficacy. However, if tumor cells develop resistance to these drugs, the induction of ICD is also weakened, subsequently affecting the anti-tumor immune response. Secondly, tumor cells can evade the immune system through various mechanisms, including downregulating antigen-presenting molecules and secreting immunosuppressive factors. These immune evasion mechanisms are sometimes linked to drug resistance, meaning resistant tumor cells might be better at evading immune recognition, complicating treatment further. Additionally, combining ICD-inducing drugs with other treatments (such as immune checkpoint inhibitors) can enhance the anti-tumor immune response and overcome some aspects of drug resistance. For instance, PD-1/PD-L1 inhibitors can lift immune suppression, making the ICD-induced anti-tumor immune response more effective. Researchers are also exploring new combination therapies and treatment strategies that simultaneously induce ICD and overcome drug resistance, including developing new drugs, targeting DAMPs signaling pathways, and designing personalized immunotherapy regimens. Overall, the relationship between ICD and drug resistance is complex and interrelated. By gaining a deeper understanding of their interactions, it is possible to develop more effective cancer therapies and improve patient outcomes.

### Ferroptosis

Regulators that induce ferroptosis may have an indirect or direct impact on GPX4 activity. To achieve this goal, several metabolic pathways are changed. This causes cells to lose their antioxidant capacity and accumulate lipid ROS. Ultimately, ferroptotic cells die from this buildup [184]. In addition, The production of reactive oxygen species (ROS) is a byproduct of the energy generation and maintenance of cancer cells' rapid proliferation, both of which are facilitated by metabolic intermediates, which are more abundant in cancer cells. In keeping with this, when exposed to high concentrations of ROS, cancer cells respond by stepping up their antioxidant defenses, thereby preventing cell death [185]. The successful use of chemotherapeutic agents, such as oxaliplatin and 5-fluorouracil, is partially linked to their ROS-generating capacities and the depletion of intracellular glutathione (GSH) [186–188]. Additionally, it has been demonstrated that exposure to iron stimulates the generation of ROS and nuclear receptor factor 2 (NRF2), which ultimately results in an increase in the expression of SLC7A11 and GPX4. This prevents iron from causing lipid peroxidation and saves colorectal cancer cells from ferroptosis [189]. When it comes to inducing ferroptosis in colorectal cancer, targeting NRF2 might therefore be a potential method. Within the realm of colorectal cancer research, investigations have revealed that focusing on ferroptosis might be a potentially fruitful treatment possibility. This has been demonstrated in vitro by the use of RSL3, which inhibits GPX4 and enhances the generation of ROS in CRC cells [190]. So far, GPX4 and SLC7A11 remain the principal targets for ferroptosis development in colorectal cancer. Several colorectal cancer studies have tested RSL3, resibufogenin, bromelain, apatinib, ACADSB, IMCA, and a slew of other ferroptosis inducers and inhibitors [191]. RSL3 inhibits GPX4 and generates ROS, which leads to a reduction in cell proliferation, which in turn leads to the suppression of CRC. IMCA reduces the development of tumors in vivo and leads to a reduction in the viability of colorectal cancer cells in vitro. This is achieved via lowering the amount of cysteine and GSH in the body, as well as reducing the expression of SLC7A11. The expression of GPX4 was negatively controlled by ACADSB, and the overexpression of ACADSB raised the levels of Fe2 + , superoxide dismutase, and lipid peroxidation in colorectal cancer cells, which ultimately led to the induction of ferroptosis [192]. Apatinib was shown to reduce the expression of GPX4 in gastric cancer [193]. Additionally, it was found to enhance ferroptosis in HCT116 cells by increasing the expression of ACSL4 and ECOVL6. This was accompanied by a decrease in the expression of GPX4 and FTH1 [194]. Contrary to the xCT inhibitors sulfasalazine and Erastin, the previously believed ferroptosis inducer sorafenib was found unable to trigger ferroptosis in many tumor cell lines [195].

**Fig. 2:**
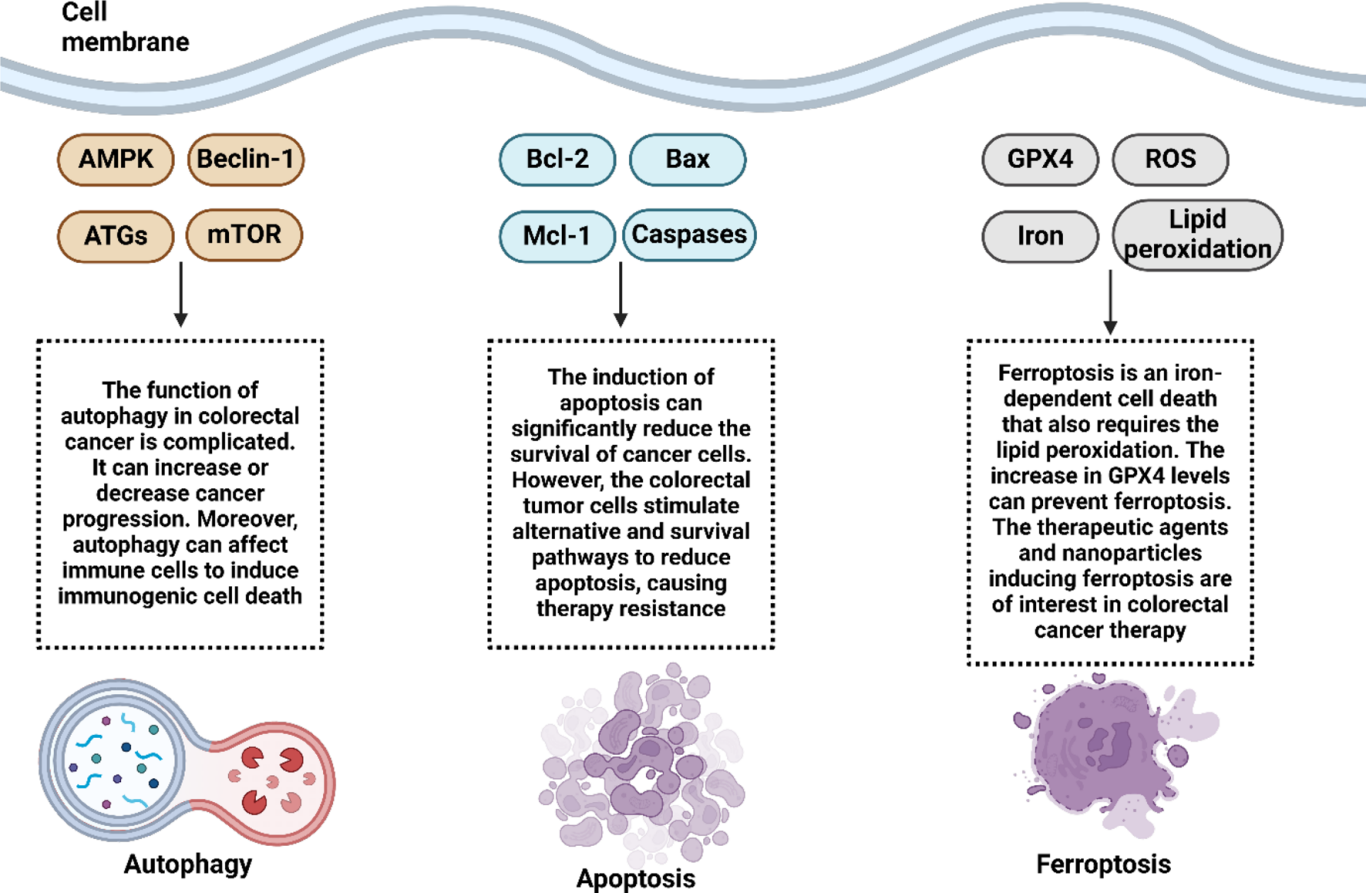
The dysregulated cell death mechanisms in colorectal cancer

The tripeptide known as glutathione (GSH) is composed of the amino acids glycine, cysteine, and glutathione. It is involved in the control of cellular activities, as well as the defense against free radicals and the metabolism of nutrients [196, 197]. Due to the loss of GSH, redox equilibrium will be disrupted, which will result in the buildup of ROS, which will ultimately lead to cell malfunction and ferroptosis [198–200]. Within the human colorectal cancer cell line (HT29-DX), it is possible to observe the correlation between enhanced GSH levels and the ability to tolerate chemotherapy [201, 202]. HT29-DX cells have greater amounts of GSH than traditional HT29 cells, which are able to accumulate doxorubicin properly [202]. HT29-DX cells are resistant to the chemotherapy drug doxorubicin, which is used to treat solid tumors. In this line, Polimeni et al. conducted research on the MRP drug-efflux pumps and discovered that HT29-DX cells exhibited considerably higher expression of MRP1 and MRP2 in comparison to sensitive controls. Furthermore, they discovered that MRP was connected with GSH-related drug resistance [202, 203]. As a result of the damage that can be caused to the GSH antioxidant defense system, cancer cells may become more susceptible to anticancer medications. As a result, adequate reductions in the amount of GSH might be beneficial to the treatment of cancer [204]. GSH insufficiency is a significant characteristic of ferroptosis, which makes therapy for colorectal cancer easier. It was discovered that the application of the cardiac glycoside oleandrin to colorectal cancer cell lines, such as SW480 and HCT116, resulted in a decrease in the concentration of GSH inside the cells and increased the rate of death in the CRC cells [205]. Xie et al. conducted a research that was quite similar to this one, in which they gave dimethyl fumarate (DMF) to several gastrointestinal cancer cell lines. This resulted in a decrease in the levels of cellular GSH, which ultimately led to the induction of apoptosis in colorectal cancer cells [206]. GPX4 is considered to be the most important factor in the regulation of ferroptosis, and GSH serves as the substrate for its activation. When it comes to cancer treatment, it is possible that directly targeting GPX4 is more successful than disrupting GSH. This is due to the fact that the cofactors of GPX4 are not restricted to GSH [207]. According to the findings of recent research, the methylation of GPX4 did not change substantially between normal cells and different types of tumor tissues. Hypermethylation of GPX4 was found to be predictive of a shorter survival rate in colon adenocarcinoma (COAD) tissues, according to research [208]. Honokiol (HNK), a bisphenol molecule, was utilized by Guo et al. [209] in order to decrease the activity of GPX4 in order to promote ferroptosis in colorectal cancer cells in human colorectal cancer cell lines. The results of this study demonstrated potential anticancer effects. GPX4 expression was shown to decrease in CNC-treated colorectal cancer cells HCT116, which suggests that CNC controls the evolution of colorectal cancer through the ferroptosis pathway [210]. Camellia nitidissima Chi (CNC) is a traditional Chinese medicine that is used to treat a variety of malignancies. By generating ferroptosis and lowering the level of GPX4 expression in CRC cells, our findings provided further confirmation that ferroptosis can be an effective treatment for colorectal cancer. In conclusion, the progression of colorectal cancer (CRC) can be controlled by the ferroptosis pathway, which offers a novel approach to the therapy of this illness. Figure 2 highlights the cell death mechanisms dysregulation in CRC.

## Colitis-associated colorectal cancer

Ever since Rudolf Virchow made the discovery of leukocyte infiltrations in neoplastic tissues in 1863 [211], inflammation has been linked to the development of cancer [212]. The dynamics of colonic epithelial cell development might be altered by inflammation, which could either promote the survival of the cells or prevent them from the process of apoptosis [213]. Multiple studies have shown a link between colon inflammation and cancer, as is the case in inflammatory bowel disease (IBD) conditions such as Crohn's disease and ulcerative colitis. The majority of inflammatory bowel disease (IBD) colorectal cancer cases involve a dysplastic precursor that transforms into a flat adenoma instead of a polyp [214, 215]. Overexpression of inflammatory genes was seen in patients with inflammatory bowel disease (IBD), including genes that are traditionally known to have a role in the development of sporadic colorectal cancer. These genes include the cyclooxygenase 2 (COX-2) genes [216] and nuclear factor kappa B (NF-κB) genes [217]. In the process of creating prostaglandin E2 (PGE2), the COX-2 enzyme plays a crucial role. PGE2 is responsible for stimulating a series of signaling pathways that ultimately contribute to the formation of tumors. These pathways include activation of phosphotidilinositol-3-kinase (PI3) and Wnt/β-catenin pathways [218]. COX-2 is expressed in forty percent of colorectal adenomas and eighty-five percent of sporadic colorectal cancers [219]. Early molecular examinations showed that colorectal tumors have a large level of mRNA (86%), whereas normal colon mucosa either has poor or nonexistent expression of this molecule [220, 221]. The high expression of COX-2 in sporadic colorectal cancer (CRC) revealed that it plays a significant role in the development of colorectal cancer, which most likely takes place at an early stage during the creation of adenomatous polyp [222]. NF-κB activation has been documented in a variety of inflammatory bowel diseases, including Crohn's disease, ulcerative colitis, self-limited colitis, and experimental colitis [223, 224]. The nuclear factor kappa B is the most significant component of the inflammatory signaling system, which has the potential to stimulate the development of tumors. A key transcription factor, it is triggered by inflammatory signals in response to infectious pathogens, cytokines, and necrotic cellular remains [225]. A transcription factor is what it is thought to be. An upregulation of genes associated with the cell cycle, apoptosis inhibitors, and proteases occurs when NF-κB is active. It is possible that certain genes could cause invasive traits. A significant difference in the expression of NF-κB was found when the immunohistochemical labeling of RelA protein, the p65 component of NF-κB, was applied to normal colorectal mucosa (9.3%), colorectal adenoma (54.0%), and colorectal adenocarcinoma (71.9%) [226].

## Hypoxia and angiogenesis

Mutations in the pathways outlined above are responsible for the development of specific cell clones that occur throughout the process of tumor initiation and promotion [227]. According to Carmelit and Jain [228], the proliferation of these cells causes an increase in the distance between individual cancer cells and blood arteries, which in turn depletes these cells of available oxygen and nutrients. During the process of tumor formation, the distances between normal cells and blood vessels are surpassed, resulting in the recruitment of new blood vessels to the tumor site through the process of angiogenesis. This is because normal cells are typically located within 100–200 μm of blood vessels owing to the diffusion limit of oxygen. Folkman [229] was the first person to identify the significance of this mechanism during the growth of tumors as well as the potential implications for cancer treatment of the disease. Our understanding of the molecular processes that underlie tumor angiogenesis has significantly expanded over the course of the last few decades, which has resulted in the discovery of VEGF-A, which is also referred to as VEGF, as one of the most important mediators of this process. Through the activation of VEGFR-1, VEGFR-2, and neuropilin 1(NP1), VEGF is able to influence the proliferation, migration, and survival of endothelial cells [230, 231]. VEGF is produced by the majority of cancer types. Furthermore, VEGF functions as a chemoattractant for haematopoietic and endothelial progenitor cells, which then integrate into the growing vasculature [232]. This process is dependent on the presence of VEGF. The production of VEGF is controlled by environmental conditions such as hypoxia or low pH, genetic alterations (such as K-ras or p53), or inflammation. Based on the findings of Ferrara et al. [231], hypoxia is considered to be the most significant trigger for VEGF expression. The first effective reduction of tumor development by the inhibition of VEGF signaling was demonstrated [233]. An experimental study using various human tumor cell lines was able to achieve this. The anti-VEFG monoclonal antibody bevacizumab was licensed for the treatment of metastatic colorectal cancer in 2004 after a phase III clinical trial showed that adding it to irinotecan, fluorouracil, and leucovorin increased survival [234]. More than a decade had passed since the therapeutic agent's initial approval when this one was handed out. Because of bevacizumab's efficacy, several medicines have been developed that target VEFG-signaling. These drugs are now undergoing clinical trials that are studying human malignancies, including colorectal cancer. Some of the drugs that fall into this category include medicines that specifically target VEFG, compounds that block VEFG receptors selectively, and multikinase inhibitors that target VEFG receptors and other kinases that contribute to tumor growth [235].

Bevacizumab, which is manufactured by Genentech and sold under the brand name Avastin, is a humanized monoclonal antibody that selectively binds to VEGF and suppresses its biologic activity by blocking it from binding to VEGFR-1 and VEGFR-2 [236]. There have been multiple clinical trials that have showed that the inclusion of bevacizumab improves clinical outcomes in metastatic colorectal cancer. The Food and Drug Administration (FDA) authorized bevacizumab as a first-line therapy for patients with metastatic colorectal cancer on February 26, 2004 [237–239]. The addition of bevacizumab to first-line chemotherapy was found to significantly enhance progression-free survival (PFS) and overall survival (OS) by 17.1 and 8.6%, respectively, according to a meta-analysis that included five randomized controlled studies. It appeared that patients who were female and those who had primary rectal tumors benefited the most [240]. However, there was a debate over the findings that were reported above in two significant studies that were part of the III series. According to the findings of the NSABP PROTOCOL c-08 study, the addition of bevacizumab to modified FOLFOX6 as an adjuvant therapy for a period of one year did not substantially increase the duration of disease-free survival in stages II and III of colon cancer [241]. According to the findings of the AVANT study, the addition of bevacizumab to adjuvant chemotherapy in patients with resected stage III colon cancer did not result in an extension of disease-free life. Additionally, the results from the OS indicate that there is a possibility of adverse effects, such as neutropenia, diarrhea, and hypertension, when bevacizumab is used with oxaliplatin-based adjuvant treatment in these patients. In comparison to the FOLFOX4 group, the bevacizumab groups experienced a significantly higher incidence of serious adverse events [242]. Prior to the administration of bevacizumab, it is imperative that major adverse effects and problems be taken into consideration. Aflibercept, also known as Regeneron, is a new recombinant fusion protein that acts as an angiogenic factor trap. It prevents the binding of VEGF-A, VEGF-B, and placental growth factor (PIGF). This fusion protein is made up of the extracellular domains of human VEGFR-1 and -2 that have been fused to the Fc region of human IgG1 [243]. Furthermore, compared to bevacizumab, aflibercept has a greater affinity for binding to VEGF-A. In addition, aflibercept has the capacity to bind to both VEGF-B and PIGF, in contrast to bevacizumab, which only binds to VEGF-A. This implies that aflibercept is capable of delivering a more comprehensive inhibition of angiogenesis i[244–246]n mCRC, The clinical trials in Phase I and II have shown that the treatment is efficacious, while also demonstrating acceptable levels of safety and tolerability. When taken in conjunction with irinotecan and fluorouracil, aflibercept showed a substantial increase in overall survival, progression-free survival, and recurrence rate (RR) when compared to placebo in a recent phase III randomized double-blind trial that was conducted on patients who had previously been treated with oxaliplatin [245]. It is interesting to note that a cost-effectiveness analysis was carried out using the Bucher technique, with hazard ratios derived from ML18147 and VELOUR. The analysis compared the treatment with bevacizumab with aflibercept in conjunction with chemotherapy [246]. According to the findings of the trials, the addition of bevacizumab to chemotherapy was shown to be just as effective as the addition of aflibercept. However, the adverse event rates and costs associated with aflibercept were significantly greater than those associated with bevacizumab plus chemotherapy. There are two endogenous inhibitors of angiogenesis that are particularly well-known: angiostatin and endostatin [247]. Both angiostatin and endostatin have the ability to attach to a wide variety of sites on the cell surface both soluble and matrix-associated. Through a number of different mechanisms, angiostatin and endostatin are able to block the proliferation, migration, invasion, and vascular morphogenesis of endothelial cells (ECs). This is accomplished by binding to integrins and other receptors that are present on ECs [248]. Based on their evaluation of the efficacy and safety of the combination of chemotherapy and Endostar, a novel recombinant human endostatin, in patients with metastatic colorectal cancer, Zhou et al. [249] came to the conclusion that the combination was well tolerated in patients with metastatic colorectal and gastric cancers, and that it was relatively effective as a first-line therapy.

The activation of the hypoxia-inducible factor (HIF) is likely the most well-studied of these cellular responses to hypoxia. According to Kizaka-Kondoh et al. [250], an HIF-1α subunit and a constitutively expressed HIF-1β subunit form the heterodimer known as the HIF-1 transcription factor. Another name for this component is aryl hydrocarbon receptor nuclear translocator (Arnt1). Modifications made after translation play a key role in controlling this heterodimer. HIF-1α is targeted for ubiquitylation by E3 ubiquitin-protein ligases and subsequent destruction through post-translational modification, which is oxygen-dependent and entails prolyl hydroxylation [251]. This is in contrast to the regulation of HIF-1α expression, which occurs through oxygen-independent mechanisms such as the PI3K- and MAPK-pathway. Therefore, in cells that are hypoxic, HIF-1α accumulates in the cytoplasm, moves to the nucleus, forms a heterodimer with HIF-1β, and binds to hypoxia-responsive regions of its target genes. Overall, this process occurs in hypoxic cells. According to Semenza [251], these genes not only comprise VEGF, which is an essential mediator of angiogenesis, but they also include genes that are involved in cell proliferation, survival, apoptosis, motility, and a great deal of other characteristics. Since it has been demonstrated that HIF-1α is overexpressed in a number of different forms of cancer, including CRC, the inhibition of HIF-1α is currently at the forefront of the list of targets for anti-tumor therapy [252]. According to Koh et al. [253], there are now a number of substances that have been reported as having the ability to block the production, degradation, and control of HIF-1α activities associated with HIF-1α. These include inhibitors of pathways that have been discussed above, such as the PI3K- or MAPK-pathway, which would result in the production of HIF-1α through their actions. EZN-2968, which is a drug that acts as an RNA antagonist of HIF-1α, has been identified by Greenberger et al. (2008). Additionally, PX-478, which is a chemical that reduces the amounts of HIF-1α protein and mRNA, has been identified by Koh et al. [253]. Additionally, both of them are now being evaluated in phase I clinical studies on a variety of solid tumors.

## Clinical implication

MSI-positive colorectal cancer is currently recognized as a separate subtype of colorectal cancer, with a stage-adjusted prognosis that is more favorable than that of individuals with MSS colorectal cancer [254, 255]. No universal agreement has been reached about the role of MSI in the adjuvant context as it pertains to the reaction to 5-fluorouracil (5-FU). It is plausible that the 5-FU adjuvant treatment may have damaged the MSI tumors rather than helping them [256]. Based on a pooled analysis of 457 newly diagnosed instances of colorectal cancer (CRC) along with the 570 CRC patients who had previously been reported, Sargent et al. (2010) confirmed the prior study's findings [257]. In contrast, the PETACC3 trial assessed the efficacy of 5-FU monotherapy in improving disease-free survival rates over 5 years for almost 600 patients with stage II and III cancer. Patients with MSI CRC had a significantly better 5-year DFS than MSS CRC patients, suggesting that the enhanced prognosis of MSI tumors was sustained during 5-FU treatment [258]. Additionally, they proved that MSI incidence varied across colorectal cancer stages II and III, and that its impact on prognosis was substantially greater in stage II compared to stage III. Beyond the 457 previously reported cases, Sinicrope et al. included 1686 patients with stage II and stage III colorectal cancer [257, 259]. Out of the total number of patients, 344 were diagnosed with multiple sclerosis. Among them, 164 had stage II CRC and 180 had stage III. Despite initial reports to the contrary, they confirmed that MSI patients fared better than MSS patients and discovered that 5-FU treatment helped patients with stage III colorectal cancer who had MSI tumors [256, 257]. It is sad that they didn't look at the impact of stage II malignancies. But they did look at how 5-FU chemotherapy affected sporadic MS vs germline MS (commonly known as HNPCC) patients. Contrary to expectations, this study found that 5-FU medication had no beneficial effects in the management of epigenetic-based sporadic MSI stage III colorectal cancer. Unlike MSI CRC, which developed due to a genetic mutation in an MMR gene, this type of cancer did not start in the body. To distinguish between germline and sporadic, the authors relied on additional parameters rather than a molecular genetic analysis of germline DNA. Consequently, further series are needed to confirm this conclusion. This need special attention, so keep that in mind. Patients with stage II/III colorectal cancer were studied in a separate adjuvant trial called the NSABP C-08. This trial examined the addition of one year of bevacizumab to oxaliplatin-based adjuvant treatment [241]. Despite a bad overall outcome, the scientists recently undertook a retrospective analysis to compare the effect of patients with MMR deficient (dMMR) cancers to those with MMR proficient tumors (pMMR). Interestingly, patients with dMMR tumors exhibited a statistically significant survival benefit (HR0.52) when administered bevacizumab, whereas those with pMMR tumors showed no benefit [260]. At the micrometastatic level, dMMR tumor cells must evade immune system attacks due to their hypermutated condition and high immunogenicity. According to their theory, this could be because VEFG-A is one of the main soluble factors produced by tumors that can establish an immune suppressive milieu. When it comes to patients with stage II colorectal cancer, for whom adjuvant treatment is not very effective, other prognostic markers than MSI have also been studied [261]. Despite the fact that several gene-expression classifiers for predicting colorectal cancer relapse have been described [262–264], such as the ColoPrint, which is an 18-gene signature [262], these classifiers are not yet routinely used in daily clinical practice. Reasons for this include small patient populations, retrospective data collection, a lack of validation sets, and the inability to use multivariate analysis to evaluate a big patient dataset. There are several clinical and future implications stemming from the genomic heterogeneity among the various serrated colorectal cancer subtypes [265–267]. Research has linked specific clinicopathological features to serrated colorectal tumors that harbor BRAF mutations, MSI, and CIMP. What an intriguing discovery: it seems that geographical location influences this molecular heterogeneity. For instance, colorectal tumors that are more common in Western populations include BRAF-mutated, CIMP, and MSI, in contrast to Eastern populations [268]. The clinical significance of MSI and CIMP genetic variations has not been fully established, in contrast to BRAF and KRAS mutations. The proximal colon is a common site for BRAF-mutated, MSI, and CIMP colorectal tumors, which disproportionately impact women [269, 270]. However, BRAF-mutated and CIMP tumors are associated with a later age of start and a poor clinical result [271, 272]. On the other hand, MSI colorectal cancers have a favorable prognosis [273]. Heavy smoking has also been linked to the development of cancers that contain a serrated BRAF mutation. When it comes to metastases, the latter appears to favor metastases in the peritoneum rather than in the liver or lungs [274, 275]. It is linked with aggressive phenotypes and has a bad prognostic result, particularly in the later stages of the illness. With or without chemotherapy, anti-EGFR treatment does not produce a response from metastatic colorectal cancers that have a BRAF mutation [276]. Furthermore, the presence of mutations in BRAF has not yet been shown to have a prognostic value for use in the treatment of colorectal cancer [270]. Even in patients with BRAF mutations who have had lung or liver metastasectomy, the 5-year disease-free survival rate and overall survival appear to be inadequate [270, 274]. As evidenced by the fact that they are involved in both the conventional and the serrated CRC pathways, KRAS mutations have a clinically diverse nature. There is a correlation between excessive body weight and cancers that carry a KRAS mutation. Although it has been suggested in the research literature, the connection between KRAS-mutated cancers and female gender is still a contentious issue [277, 278]. In metastatic colorectal cancer, KRAS-mutated tumors have been primarily investigated. These tumors have been linked to a bad result, specifically a low 5-year disease-free survival. However, they have also been linked to a short cancer-specific survival in stage I colorectal cancer [266, 279]. The presence of KRAS mutations in colorectal cancer shows resistance to anti-EGFR treatments as well as a poor response to therapies based on 5-fluorouracil [280]. The development of metastatic disease can also be accelerated by the presence of KRAS mutations in conjunction with MSS [281]. A good prognosis is often indicated by MSI, which is a molecular marker that is independent of the status of BRAF, KRAS, and CIMP [266]. Multiple sclerosis is also linked to a decreased incidence of disorders that are in their later stages [282, 283]. Cancer patients who have MSI tumors often have a more favorable prognosis and a longer disease-free life compared to those who have MSS tumors [284]. Additionally, treatment is beneficial for MSI cancer patients [285]. The survival rate for individuals with metastatic MSI colorectal cancers who have BRAF mutations is, however, rather low [286]. In order to assess chemotherapy-based regimens, the MSI test is indicated for patients who have achieved stage II of colorectal cancer [287]. However, the therapy with 5-fluorouracil does not have a beneficial effect on survival rates in individuals who have MSI colorectal cancer [288].

The accumulation of data implies that certain microRNAs (miRNA) have the power to modulate MMR expression, which in turn can alter genomic stability in colorectal cancer [289]. It has been demonstrated that the downregulation of MMR proteins and the induction of MSI in colorectal cancer cells may be achieved by the ectopic expression of either miR-155 or miR-21 [290, 291]. Overexpression of miR-155 or miR-21 was found to have an adverse relationship with the degree of hMLH1 and/or hMSH2 protein expression in human colorectal tumors [291, 292]. In addition, an overexpression of miR-155 was discovered in a subgroup of tumors that had an unidentified contributing factor to MMR inactivation [292]. Overexpression of miR-21 was shown to significantly limit the effectiveness of 5-FU in a colorectal cancer xenograft model, and this was found to be connected with a decrease in the expression of hMSH2 [291]. Taking all of these early findings into consideration, it appears that these microRNAs may play a role in the etiology of MSI and may also serve as a possible indication of the 5-FU response. According to recent findings, the molecular heterogeneity that is present in MSI colorectal tumors has been expanded. Both MSI cell lines and human colorectal tumors were discovered to have a mutation in the gene that codes for heat shock protein (HSP) 110 [293]. There was no evidence of chaperone functions or antiapoptotic effects in the HSP110 shortened protein, which is characteristic of HSPs. There was an indication of survival advantage in two small retrospective cohorts of MSI colorectal tumors treated with adjuvant 5-FU with or without oxaliplatin [293]. This mutation was found to sensitize MSI colorectal cancer cells to therapy with 5-FU and oxaliplatin, despite the fact that it was of a very preliminary nature. MSI cancers were shown to be related with greater frequencies of inactivation of the PTEN tumor suppressor gene due to mutation or hypermethylation in comparison to MSS tumors in prior research [294, 295]. Sporadic multiple sclerosis in the colon (MSI) colon tumors that have epigenetic inactivation of MLH1 exhibit a high frequency of BRAFV600E mutations, around fifty percent, in comparison to the general BRAF mutation frequency of eight to eleven percent among colorectal malignancies [296, 297]. A serine/threonine kinase that is an important component of the RAF/MEK/ERK signaling cascade is encoded by the BRAF gene [298, 299]. Specifically, BRAF mutations are found in a hotspot in exon 15 of colorectal tumors, which ultimately results in a single-amino-acid replacement of the V600E position [298]. There is a mutually exclusive relationship between BRAF mutations and KRAS mutations, which are more frequently linked with patients who have MSS tumors [299]. The presence of a BRAF mutation is indicative of sporadic multiple sclerosis of the colon (MSI) colorectal cancer and, in essence, eliminates the possibility of a Lynch syndrome diagnosis [300]. When it comes to colorectal malignancies, BRAFV600E mutations have been linked to a more dismal prognosis throughout all stages of the tumor [297, 301]. Recent reports, on the other hand, have provided contradictory information about individuals with stage II/III colon cancer who are taking part in adjuvant chemotherapy studies. To be more specific, the BRAF mutation was not related with a prognostic value in stage II tumors in the QUASAR adjuvant study [302], but it was associated with a decreased overall survival (but not recurrence-free survival) in stage II/III cancers in the PETACC-3 adjuvant trial [303]. It is not yet known if BRAF mutations can provide prognostic information within the subgroup of MSI tumors [304], but this is something that has to be addressed. In summary, MSI status is a crucial factor in the prognosis and treatment response of colorectal cancer. The heterogeneity in genetic mutations like BRAF and KRAS, along with microRNA regulation, underscores the need for personalized treatment strategies. Further research and clinical trials are necessary to refine these approaches and incorporate them into routine practice.

## Prognostic factors

In order to determine whether or whether the molecular features of colorectal carcinomas are beneficial in determining the prognosis, research is now being conducted [305]. Over the course of a trial that involved 662 patients, Ogino and colleagues discovered that individuals who had tumors that had greater amounts of COX2 were associated with a worse survival rate [306]. In addition, the research conducted by Soumaoro and colleagues shown that there is a positive link between higher COX2 levels and a worse prognosis. According to the findings of this investigation, COX-1 levels did not appear to have any impact on the prognosis [307]. A high death rate has also been shown to be associated with BRAF mutations, according to a second study that was conducted by Ogino and colleagues [301]. In addition, research conducted by Malesci and colleagues suggests that tumors that have MSI has a lower probability of being related with distant metastasis [308]. Deletions of the long arm of chromosome 18 have been discovered in more than seventy percent of colorectal tumors. These deletions typically affect the DCC gene, which has been demonstrated in certain studies to correlate with metastasis and a poor prognosis. However, confirmation by prospective studies is still missing [309]. There have been a number of big investigations, the most notable of which being the RASCAL I and II studies, which have shown that there is a correlation between KRAS mutations and a more catastrophic outcome. It has been proven that the prognosis is affected differently by various mutations. The existence of a mutation in the KRAS gene that changes the codon 12 from glycine to valine in Duke's stage C tumors has been linked to an aggressive behavior of the tumors. Additionally, mutations in codon 13 have been linked to a mortality risk that is forty percent higher than previously thought [310].

There is currently a dearth of clear data in the form of big prospective trials, despite the fact that studies have shown that having MSI is associated with a better prognosis. This is in part owing to the fact that it is difficult to perform such research since spontaneous instances of colorectal cancer that are positive for MSI occur only at a very low frequency [311]. Patients with MSI tumors have a better prognosis than patients with MSS tumors, according to the findings of a systematic analysis that was conducted by Popat et al. (2005). The research included data from 32 different studies that included a total of 7642 patients [283]. Given these findings, it is possible that the MSI status might be utilized in order to identify individuals who have the potential to be treated only by surgical intervention. The response of MSI tumors to 5-fluoruracil (5-FU) appears to be ineffectual, whereas the effectiveness of the response varies depending on the stage of the tumor [312]. The capacity to anticipate individual patient responses to different chemotherapeutic treatments would be a desired outcome that would result from the study that is being conducted into the molecular pathways leading to the development of colorectal cancer. In point of fact, a significant amount of research has been conducted in this field, which has generated the prospect that patients may be classified as either responders or non-responders to adjuvant chemotherapy administration [313]. Today, individuals who have metastatic illness frequently get treatment with monoclonal antibodies that are targeted against EGFR. It has been demonstrated that the responsiveness of a person to anti-EGFR therapy can be determined by certain mutations in the KRAS gene [314]. Patients who have been diagnosed with a primary tumor that has the wild type KRAS gene are the only ones who should be considered for anti-EGFR therapy, according to the recommendations made by European authorities.ten Although there is some evidence that BRAF mutations, most notably V600E, have a role in predicting how well anti-EGFR treatment will work, this data is limited. It is possible that the negative association that exists between the V600E BRAF mutation and the response to anti-EGFR therapy might serve as a foundation for the further subdivision of wild type KRAS positive tumors of patients [315]. Adjuvant chemotherapy also targets Topoisomerase I (topo-I), which is inhibited by the camtothecin analogue irinotecan. Topo-I is another target for adjuvant chemotherapy. Through the process of nicking and re-ligating the DNA strand, Topo-I is able to operate onto supercoiled DNA strands and assist in the relaxing of the DNA strand prior to the replication fork. As a result, it is an important component in the process of DNA replication. While non-randomized clinical trials have demonstrated a favorable correlation between topo-I levels and irinotecan sensitivity, they have failed to prove a conclusive connection. This association has already been shown in trials performed on cell lines. In contrast, a major randomized trial by Braun et al. (2008) found that topo-I levels were predictive of a good response to irinotecan or oxaliplatin. In addition, it was demonstrated that these levels serve as a prognostic indicator for 5-FU therapy [316].

## Conclusion

After forty years of research, scientists finally have a firm grasp on the genetic and epigenomic instability that plays a role in the progression of colon polyps to colorectal cancer [317]. One of the initial successes of the precision medicine era was the use of KRAS-mutation analysis to direct anti-EGFR medication. It has become one of the most successful approaches in the field, producing biomarkers that have been satisfactorily confirmed for routine clinical use in the management of CRC. To prioritize molecular genetic testing for Lynch syndrome, MSI and BRAF mutations are now important. For sporadic colorectal tumors, these markers are likewise expected to have a far larger impact on prognosis and therapeutic response prediction. The recent success of methylation VIM, NDRG4, and BMP3 as early detection tests for CRC suggests that epigenetic modifications could be useful molecular indicators in a clinical setting. While there is some potential in using molecular abnormalities to predict the risk of metachronous polyps or CRC, additional study is needed to determine whether abnormally methylated CpGs or other molecular alterations can be used as accurate and reliable markers of polyp or CRC risk. Because there may be no agreement in the polyps regarding patterns of genomic instability or specific methylation sites, a method focused on markers inside the polyps themselves may not work. On the other hand, if a field cancerization impact is present, it can be helpful to evaluate the normal mucosa. Researchers over the next five to ten years should be able to put these questions to rest and find new clinical uses for molecular anomalies in colon polyps and colorectal malignancies, as is widely believed. The exploration of CRC through the lens of cellular and molecular biology has unveiled a complex landscape of genetic and epigenetic alterations, signaling pathway dysregulations, and aberrant cell death mechanisms. This comprehensive study has highlighted the crucial role of key molecular pathways such as Wnt, EGFR/MAPK, and PI3K in the oncogenesis and progression of CRC. Importantly, the dual nature of autophagy in CRC, functioning both as a tumor suppressor and a promoter, underscores the intricate balance between cell survival and death mechanisms in cancer biology. Our findings underscore the significance of genetic predispositions, environmental influences, and their interplay in CRC risk and development. Moreover, the identification of specific molecular markers and pathways not only enhances our understanding of CRC pathogenesis but also opens new avenues for targeted therapeutic interventions. The potential of these molecular factors as diagnostic and prognostic tools offers hope for more personalized and effective management of CRC. In conclusion, the battle against colorectal cancer, a formidable adversary in oncology, requires a multifaceted approach that includes advanced understanding of its molecular underpinnings, early detection strategies, and the development of targeted therapies. As research continues to unravel the molecular complexities of CRC, it promises to guide the development of novel diagnostics and therapeutics, ultimately improving patient outcomes and survival rates. The journey from understanding to cure is arduous and requires persistent efforts in research, but it is through such comprehensive studies that we inch closer to conquering colorectal cancer.

## Data Availability

The datasets generated during and/or analyzed during the current study are available from the corresponding author upon reasonable request.
